# Strengths of pelvic floor muscles in women with multiple sclerosis and its relationship with urinary incontinence and quality of life

**DOI:** 10.3389/fneur.2024.1514157

**Published:** 2025-01-13

**Authors:** Poorandokht Afshari, Parvin Abedi, Nastaran Majdinasab, Samaneh Tafakh, Mohammadhossein Haghighizadeh

**Affiliations:** ^1^Midwifery Department, Nursing Care Research Center in Chronic Diseases, Ahvaz Jundishapur University of Medical Sciences, Ahvaz, Iran; ^2^Midwifery Department, Menopause Andropause Research Center, Ahvaz Jundishapur University of Medical Sciences, Ahvaz, Iran; ^3^Department of Neurology, School of Medicine, Musculoskeletal Rehabilitation Research Center, Ahvaz Jundishapur University of Medical Sciences, Ahvaz, Iran; ^4^Midwifery Department, Menopause Andropause Research Center, Ahvaz Jundishapur University of Medical Sciences, Ahvaz, Iran; ^5^Biostatistics Department, Ahvaz Jundishapur University of Medical Sciences, Ahvaz, Iran

**Keywords:** multiple sclerosis, pelvic muscle strengths, urinary incontinence, quality of life, reproductive age

## Abstract

**Background:**

Multiple sclerosis (MS) is a debilitating autoimmune disease that mostly affects women.

**Objectives:**

In this study we evaluated the relationship of pelvic muscle strengths with urinary incontinence and quality of life in women with MS.

**Materials and methods:**

In this cross-sectional study 87 women with MS were recruited. Data collected using a demographic questionnaire, the International Consultation on Incontinence Questionnaire-Urinary Incontinence Short Form (ICIQ-UI SF), and Quality of Life Questionnaire (QOL-SF-36). A perineometer was used to measure the strengths of pelvic muscle. Data analyzed using Pearson correlation test, and multiple linear regression tests.

**Results:**

There was a positive correlation between pelvic muscle strengths with all domains of quality of life except for body pain and role limitations due to emotional problems. A significant inverse correlation was found between urinary incontinence and all domains of quality of life except for body pain. Also, an inverse correlation was found between muscle strength and urinary incontinence (*r* = −0.838, *p* < 0.001). A one-unit increase in the quality of physical life was associated with a 0.15-unit increase in the strengths of pelvic floor muscles (*p* = 0.035). On the other hand, each additional year of marriage or disease duration significantly weakened pelvic floor muscles by 0.24 and 0.509 units, respectively (*p* < 0.05).

**Conclusion:**

Our findings showed that pelvic muscle strength, urinary incontinence, and quality of life were significantly interrelated among female patients with MS. An inverse correlation was also found between muscle strength and urinary incontinence. Duration of marriage and length of MS disease were inversely associated with the strength of the pelvic floor muscles. Health providers are recommended to educate MS patients on the importance of pelvic muscle strengths.

## 1 Introduction

Multiple sclerosis (MS) is a debilitating autoimmune chronic disease in which the immune system of the body attacks the myelin of nerve cells, causing permanent damage to nerves (1). Although a combination of genetic and environmental factors such as viral illnesses or stress can trigger the onset of the disease, the underlying cause of MS is still unknown (2). Epstein–Barr virus (EBV) infection, ultraviolet B light, obesity, vitamin D insufficiency and deficiency, and smoking are among the environmental factors that cause the disease to flare up (3).

The estimated rate of people affected with MS is 2.8 million around the world, of whom nearly a million live in America (4). A study on data obtained from Global Burden of Disease (GBD) in 2019 showed that 59,345 cases affected with MS, and 22,439 deaths happened due to this disease (5).

Women are three times more likely to develop MS compared with men (6). The prevalence of MS in the Iranian population has been reported to be 30 per 100,000, with a higher prevalence (44.8 per 100,000) among women (7).

MS is an unpredictable disease that presents with different signs and symptoms. The main factor that causes neurological symptoms in this disease is a decrease in the myelin of nerve endings in the brain and spinal cord, and therefore neuronal death and the neurological symptoms are much prevalent (8). The most prevalent symptoms include low vision, weak muscle reflex, movement difficulties, and decreased co-ordination (9).

Women with MS experience more stress urinary incontinence (SUI) and urgency urinary incontinence (UUI), both of which restrict their physical activity and negatively affect their quality of life (10). A systematic review of 12 studies showed that the prevalence of lower urinary tract symptoms was 68.4%, with increased urinary frequency as a dominant problem (11). A cross-sectional study on 602 Iranian MS patients showed that the prevalence of lower urinary tract symptoms was 87.6%. While this range did not show any difference between men and women, SUI was more prevalent among women than men (12).

The causes of urinary problems in women with MS may be attributed to detrusor overactivity, voiding dysfunction, and bladder involuntary contractions (13). The potential role of pelvic muscle weakness in urinary problem, however, is not clear. Some evidence shows that MS does not affect pelvic muscles (14). Contrary to this evidence, Lucio et al. in their study on MS women with lower urinary tract problems found that a muscle training intervention could significantly decrease overactive bladder, voiding symptoms, and stress incontinence, which will improve the quality of life (15). Also, the negative effect of urinary problems such as overactive bladder on quality of life has been proven in a previous study (16).

### 1.1 Research question

Given the limited research in the relationship between pelvic muscle strength, urinary incontinence, and quality of life, we aimed to investigate this association in Iranian women with MS. Our hypothesis was that reduced pelvic muscle strength in women with MS would lead to urinary incontinence and decrease quality of life.

## 2 Materials and methods

This cross-sectional study included 87 reproductive-aged women with MS. All consenting women with MS disease were recruited, excluding single women and those with severe physical disabilities. MS diagnosis was confirmed using the 2017 Revised McDonald criteria (17). Data collection started in March 2020 and completed in September 2020.

### 2.1 Ethics approval and consent to participate

The Ethics Committee of Ahvaz Jundishapur University of Medical Sciences approved the design of the study (Ref ID: IR.AJUMS.REC.1395.518). Before data collection written informed consent was obtained from all eligible women. All procedures followed were in accordance with the ethical standards of the responsible committee on human experimentation (institutional or regional) and with the Declaration of Helsinki 1975, as revised in 2013.

### 2.2 Setting

We screened women according to inclusion/exclusion criteria in an MS clinic (located in the Rehabilitation Faculty of Ahvaz Jundishapur University of Medical Sciences) in Ahvaz, Khuzestan province, southwest of Iran. This center registers all MS patients in Khuzestan province. At time of data collection, 2,300 individuals suffering from MS were registered in the MS society of Khuzestan, of whom 400 were reproductive-aged women living in Ahvaz. Two hundred women were involved in other research projects, and only 87 out of the 200 remaining women provided consent to participate in the present study.

### 2.3 Data collection methods

Data collection instruments were as follows: a demographic questionnaire, the International Consultation on Incontinence Questionnaire-Urinary Incontinence Short Form (ICIQ-UI SF), and the Quality of Life Questionnaire (QOL-SF-36). The demographic questionnaire inquired about age, length of marriage, number of pregnancies, body mass index, length of disease, marital status, mode of birth, educational attainment, occupation, and disease relapse were incorporated in the demographic questionnaire.

Urinary incontinence during the past 4 weeks was assessed using ICIQ-SF questionnaire. Two initial questions (Question 1 and 2) were about demographic characteristics (age and sex), Questions 3, 4, 5 and 6 were about frequency of urinary incontinence, urinary leakage, effect of urinary incontinence on quality of life, and the time of urinary leakage, respectively. The total score of this questionnaire is between zero and 21, with higher scores indicating worse urinary incontinence (18). Hajebrahimi et al. (19) validated this questionnaire in Iran.

The quality of life of the participants was assessed using QOL-SF-36. This questionnaire has 36 questions and 8 subscales. The subscales include physical function (10 questions), role limitations due to physical problems (4 questions), vitality, energy/fatigue (3 questions), social functioning (2 questions), role limitations due to emotional problems (3 questions), bodily pain (2 questions), general mental health (6 questions), and general health perception (6 questions). Each subscale is scored from zero to 100, with higher scores indicating better quality of life. This questionnaire has two major domains including the physical component summary (PCS) and the mental component summary (MCS) (20). This questionnaire was validated by Montazeri et al. (21) in Iran.

### 2.4 Procedures

Eligible women were requested to complete the three questionnaires. In case the women had any question, one of the researchers (ST) was available to help them.

To measure the strengths of pelvic muscle, the women were first placed in the lithotomy position. A perineometer covered by a plastic cover (condom) was placed in the vagina. Then, women were requested to contract their vagina and the perineum with maximal effort. The peak force reading on the device was recorded, and this procedure was repeated two more times. The average of three measurements was recorded as final pelvic muscle strengths. All measurements were conducted by a single trained researchers (ST).

The weight of participants was measured using a digital scale (Seca, Germany made) while they were barefooted and wearing light clothing. The height of the participants was measured using a stadiometer while they were standing barefooted. Body mass index (BMI) was calculated using a formula of weight (kg)/height (m^2^).

### 2.5 Data analysis

Qualitative data were reported using frequency and percentages while quantitative data were reported using mean and SD. Pearson Correlation coefficient was used to examine the correlation of pelvic muscle strengths with urinary incontinence and quality of life. The relationship between pelvic muscle strengths, stress incontinence, and quality of life were assessed using multiple linear regression (adjusting for confounding variables). SPSS version 25 was used for data analysis. *p* < 0.05 was considered statistically significant.

## 3 Results

According to our results, most of the participants were of the reproductive age and had been married for 10 years. The average number of pregnancies was two, and the mean of their BMI was 26.38 ± 2.32. The duration of MS disease was around 5 years, and the distribution of normal vaginal delivery and cesarean section were similar among participants. Most women (42.5%) were housewives with secondary high school education. The economic status of most participants was moderate. While most participants (50%) had no recurrence of the disease, 43.7% reported at least one recurrence (Table 1).

**Table 1:** Characteristics of women with MS disease.

Variables	MS patients *N* = 87
	Mean ± SD
Age (y)	34.86 ± 6.74
Duration of marriage (y)	11.37 ± 8.44
Number of pregnancies	1.79 ± 1.67
Body mass index (kg/m^2^)	26.38 ± 2.32
Duration of disease (y)	4.59 ± 3.89
	N (%)
Marriage status	
Married	82 (94.3)
Single	5 (5.7)
Type of birth	
Nulliparous	17 (19.5)
Normal vaginal delivery	19 (21.8)
Vaginal delivery + Episiotomy	24 (27.6)
Cesarean section	26 (29.9)
Education	
Primary	5 (5.7)
High school	12 (13.8)
Secondary high school	37 (42.5)
University	33 (37.9)
Occupation	
Employed	29 (33.3)
Housewife	58 (66.7)
Economic status	
Poor	3 (3.4)
Moderate	66 (75.9)
Good	18 (20.7)
Disease relapse	
No	44 (50.6)
Once	38 (43.7)
Twice	2 (2.3)
>twice	3 (3.4)

The mean score of pelvic muscle strength was 21.03 ± 8.45. Data revealed that women who had normal vaginal delivery had the highest perineal strength (23.91 ± 9.12) followed by those women who underwent cesarean section (21.65 ± 7.95). Women who had normal vaginal delivery plus episiotomy had the weakest perineal muscle strength (16.68 ± 5.66) (Data are not shown in Tables).

The characteristics of urinary leakage in the study participants are illustrated in Table 2. A substantial number of women (63.2%) reported urinary leakage, which mostly (57.5%) happened with sneezing. Also, a considerable proportion (33.3%) reported that they experienced urinary leakage three times a week.

**Table 2:** Urinary leakage in the study participants (*N* = 87).

Variables	N (%)
Urinary leakage	
No leakage	32 (36.8)
Once per week	14 (16.1)
3 times per week	29 (33.3)
Once per day	5 (5.7)
Always	7 (8)
How do you estimate the amount of urinary leakage?	
No leakage	32 (36.8)
Low	16 (18.4)
Moderate	25 (19.5)
High	14 (16.1)
Leakage with sneezing	50 (57.5)
Leakage at night	20 (23)
Leakage during physical activity	33 (37.9)
Leakage after urination	5 (5.7)

The scores of quality-of-life domains are presented in Table 3. As this table shows, the lowest scores were related to role limitations due to the emotional problems, physical functioning, and general health (43.65 ± 32.17, 48.51 ± 25.97, and 46.85 ± 13.66, respectively), while role limitations due to physical health had a higher score (67.60 ± 17.71).

**Table 3:** The scores of quality of life and its subscales in women with multiple sclerosis (*N* = 87).

Variables	Mean ± SD	Median	Minimum	Maximum
Role limitations due to emotional problems	43.65 ± 32.17	33.33	0.00	100
Energy/fatigue	53.31 ± 14.52	55.00	20.00	85.00
Emotional well-being	57.77 ± 13.01	56.00	24.00	92.00
Social functioning	61.11 ± 17.03	62.50	12.50	100
Physical functioning	48.51 ± 25.97	50.00	0.00	100.00
Role limitations due to physical health	67.60 ± 17.71	70.00	30.00	95.00
General health	46.85 ± 13.66	45.00	25.00	90.00
Body Pain	62.26 ± 15.62	67.50	22.50	97.50
Physical domain	56.31 ± 13.40	57.50	22.50	85.63
Psychological domain	53.96 ± 15.28	51.83	18.13	91.13
Total score of quality of life	55.13 ± 12.62	55.54	26.44	81.56

The Pearson Correlation coefficient was used to assess the correlation of pelvic muscle strength with urinary incontinence and quality of life. As Table 4 shows, pelvic muscle strength had a positive correlation with all domains of quality of life except for pain and role limitations due to emotional problems. There was an inverse significant correlation between urinary incontinence and all domains of quality of life except for pain (Table 4). An inverse correlation was also found between muscle strength and urinary incontinence (*r* = −0.838, *p* < 0.001) (Data are not shown in table).

**Table 4:** Relationship between pelvic muscle strengths, urinary incontinence and quality of life.

Variables	EF	EW	SF	RE	PF	RP	GH	Body pain	Physical	Mental	Total quality of life
Strengths of pelvic muscles	0.440	0.420	0.244	0.206	0.331	0.358	0.439	0.207	0.450	0.372	0.465
*p* value	<0.001	<0.001	0.023	0.056	0.002	0.001	<0.001	0.055	<0.001	<0.001	<0.001
Urinary incontinence	−0.55	−0.455	−0.337	−0.267	−0.216	−0.350	−0.439	−0.163	−0.380	−0.462	−0.481
*P* value	<0.001	<0.001	0.001	0.012	0.045	0.001	<0.001	0.131	<0.001	<0.001	<0.001

EF, Energy/fatigue; EW, Emotional well-being; SF, Social functioning; RE, Role limitations due to emotional problems; PF, Physical functioning; RP, Role limitation due to physical health; GH, General Health.

The effect of physical and mental dimensions of quality of life on the strengths of pelvic floor muscles was assessed using the multiple linear regression (Table 5). As results indicated, a one-unit increase in the quality of physical life was associated with a 0.15-unit increase in the strength of pelvic floor muscles (*p* = 0.035). On the other hand, durations of marriage and the disease were inversely related to the strengths of the pelvic floor muscles. Also, a one-year increase in marriage duration was associated with a 0.24-unit decrease in strength (*p* = 0.027), while a one-year increase in disease duration was associated with a 0.509-unit decrease (*p* = 0.025). A one-unit increase in the number of normal vaginal deliveries was associated with a 4.072-unit increase in pelvic muscle strength, although this relationship did not reach statistical significant (*p* = 0.085).

**Table 5:** Multiple linear regression for assessing pelvic muscle strength and urinary incontinence.

Pelvic muscle strengths	Coef. (SE)	t	*P* value	95% Confidence interval		R-square
				Lower	Upper	
Marriage duration	−0.240 (0.107)	−2.25	**0.027**	453–0.	−0.028	0.414
Length of MS disease	−0.509 (0.222)	−2.29	**0.025**	−0.951	−0.067	
NVD + Epi	−1.386 (2.505)	−0.55	0.582	−6.374	3.602	
C/S	1.625 (2.334)	0.70	0.488	−3.022	6.272	
NVD	4.072 (2.330)	1.75	0.085	−0.567	8.711	
Physical domain	0.146 (0.068)	2.15	**0.035**	0.011	0.281	
Mental domain	0.064 (0.058)	1.11	0.270	−0.051	0.179	

NVD, Normal vaginal delivery; C/S, Cesarean section; Epi, Episiotomy. The bold *P* values means significant.

## 4 Discussion

This study evaluated the relationship of pelvic muscle strength with urinary incontinence and quality of life in Iranian female MS patients. The results of this study indicated that the strengths of pelvic muscles decreased in these patients, with 63.2% of them reporting urinary leakage happening with sneezing.

Similar to our findings, Zeca et al. (22) reported a 35% prevalence of urinary incontinence in 403 MS patients, predominantly female (72%), with higher prevalence in disabled and female patients. Another study conducted in Iran on MS patients showed that the prevalence of overactive bladder among MS patients was 36.4% (23), which is much lower than that obtained in the present study. This discrepancy may be because in the present study we did not measure overactive bladder and we enrolled only female patients with MS disease, while in the above-mentioned study, both male and female patients were recruited.

In line with our results, Murphy et al. in their study on 143 female MS patients, found that 55.9% of their patients had stress urinary incontinence, 70.6% had urgency urinary incontinence, and 44.8% had mixed urinary incontinence (10). Our data showed an inverse correlation between muscle strength and urinary incontinence in MS patients. These results were confirmed by Mubaraki et al. (24) in their study on 150 MS patients, the results showed that the prevalence of urinary incontinence and overactive bladder were significantly higher in MS patients with progressive MS patients.

Our results showed that pelvic muscle strengths had a positive correlation with all domains of quality of life except for pain and role limitations due to emotional problems. Furthermore, urinary incontinence had a reverse significant correlation with all domains of quality of life except for pain. Evidence show that the performance and the strength of core muscles are significantly decrease in MS patients (14). Also, depression, fatigue, and low quality of life are prevalent among MS patients, which be improved if they have regular physical activity (25). Lucio et al. (15) found that both the strength of perineal muscles and the quality of life decreased in female patients with MS, and the urinary incontinence is prevalent among them. They showed that perineal muscle training for 12-week could significantly improve the overactive bladder, urinary incontinence, and quality of life.

The results of multiple linear regression in our study showed that by one-unit increase in the quality of physical life, the strength of pelvic floor muscles increased by 0.15 units. Other studies evaluated the relationship of physical functioning with pelvic muscle strength. For instance, McClurg et al. (26) studied 37 patients with MS disease and found that 9 weeks of pelvic muscle training using electromyography biofeedback could significantly increase pelvic muscle strengths and reduce the urinary incontinence. In their study, Mahajan et al. (27) on 9,397 patients with MS disease found that 41, 41, and 42% of females had urinary, bowel, and sexual problems which are all related to pelvic muscle weakness. Our results are similar with those of McClurg et al. (26) and Mahajan et al. (27).

Our results showed that with increasing the duration of marriage and of disease duration, the strength of the pelvic floor muscles was decreased. The reason for this matter is women with longer duration of marriage obviously are older and evidence shows that the motor discharge rate and muscle size decreased in MS patients, which might worsen with aging if patients do not have regular physical activity (28). The structural and functional changes in MS disease may resulted from changes in primary motor cortex (M1) in the cortical areas, that may enhance with using neuromodulatory techniques (29). A recent study showed that MS women need more time for rehabilitation using electrotherapy, that it may contributed to the weakness of muscle in women (30).

Our results showed that women with normal vaginal delivery had the highest pelvic strength followed by women who underwent cesarean section. Women who had normal vaginal delivery plus episiotomy had the weakest pelvic muscle strengths. Also, results of multiple linear regression showed that the mode of delivery does not have a significant effect on the strength of the pelvic floor muscles. However, according to the regression model, it seems that if the sample size was larger, women with normal vaginal delivery will show stronger pelvic muscles.

Although in the present study, we did not have a control group, we conducted a study on 341 healthy women in 2017 and measured the strengths of their pelvic muscles. The results showed that the highest pelvic muscle strengths were belong to nulliparous (55.62 ± 15.86 cm H2O) followed by women who had vaginal delivery with intact perineum (53.88 ± 20.9), women who underwent elective cesarean section (52.9 ± 21.29), and those underwent an emergency cesarean section (48.97 ± 21.04). Results indicated that women who had vaginal delivery with episiotomy had the weakest pelvic strength (32.71 ± 14 cm H2O) (31). Results of the present study are almost in line with the results of a study we did on healthy women, except for the fact that the pelvic muscle strength of women with MS is about half of that of healthy women.

However, other studies reported conflicting results. For example, Fang et al. in their study on 21,302 participants found that women with elective cesarean section had significantly rapid, tonic, and endurance contractions of perineal muscles compared to vaginal delivery (32). Furthermore, Jansson et al. (33) in a study found that women with vaginal delivery experienced more stress incontinence 1 year after childbearing. Both above-mentioned studies did not mention about whether women with vaginal delivery underwent episiotomy, and there was not results of relationship of parity and muscle strengths or urinary incontinence.

### 4.1 Strengths and limitations of study

This is the first time that we evaluated the strengths of pelvic muscle with urinary incontinence and quality of life among Iranian women with MS disease. This study has some limitations. First, we did not recruit women randomly, and only 87 women who gave consent were recruited in this study, which may affect generalizability of the results. Second, we did not have a control group, that may limit the ability to contextualize the observed reduction in pelvic muscle strengths compared with healthy women. And finally, we did not assess the bowl function of women.

## 5 Conclusion

The results of this study showed that pelvic muscle strengths decreased in the women with MS disease, and it had a positive correlation with all domains of quality of life except for pain and role limitations due to emotional problems. Also, urinary incontinence had a reverse significant correlation with all domains of quality of life except for pain. An inverse correlation was also found between muscle strength and urinary incontinence. Pelvic floor muscle strength was inversely related to the duration of marriage and the length of MS disease. Health providers are recommended to educate MS patients about the importance of pelvic muscle strength.

## Data Availability

The raw data supporting the conclusions of this article will be made available by the authors, without undue reservation.
